# Use of the Ion Robot in the Diagnosis of Pulmonary Nodules: Fine Needle Aspiration Versus Cryobiopsy

**DOI:** 10.1002/dc.70136

**Published:** 2026-04-29

**Authors:** Suzanne M. Selvaggi

**Affiliations:** ^1^ Department of Pathology and Laboratory Medicine University of Wisconsin School of Medicine and Public Health Madison Wisconsin USA

**Keywords:** cryobiopsy, diagnostic yield, fine needle aspiration, ion robot, sensitivity, specificity

## Abstract

**Background:**

Robotic‐assisted bronchoscopy platforms provide an innovative approach to the sampling of pulmonary nodules. As compared to other technologies, fiber‐optic shape‐sensing instrumentation allows for a more precise, accurate location and sampling of the target lesion with fewer complications. As FNA is a mainstay in the evaluation of pulmonary nodules, this study compares FNA diagnostic yield to cryobiopsy using the Ion robotic system.

**Methods:**

From January 1, 2025 through June 30, 2025, the Cytopathology laboratory processed 87 FNAs with concurrent cryobiopsies from pulmonary nodules and form the basis of this study.

**Results:**

Of the 87 FNAs, 45 (52%) were positive/suspicious for malignancy, 4 (5%) were positive for a neoplasm, 5 (6%) were atypical, 13 (15%) were negative and 20 (23%) were nondiagnostic. Cryobiopsies were concordant in 59 (68%) cases and discordant in 28 (32%) cases. Of the 28 discordant cases, 3 had neoplastic cells on FNA of which 2 were nondiagnostic and 1 showed atypical cells on cryobiopsy. Of the 5 FNAs with atypical cells, 5 were malignant on cryobiopsy. Of the 20 nondiagnostic FNAs, 13 were malignant and 7 were negative on cryobiopsy. FNA diagnostic yield was 71%, sensitivity 66%, and specificity 100%. The cryobiopsy diagnostic yield was 96%, sensitivity 94%, and specificity 100%.

**Conclusion:**

At our institution, use of the Ion robotic system for the evaluation of pulmonary nodules has led to increased diagnostic yields over standard FNA with ROSE. This technology provides an additional modality for the sampling of pulmonary nodules; in particular, those located peripherally.

## 1 Introduction

The Ion Endoluminal System, developed by Intuitive Surgical Inc., is a robotic‐assisted system for minimally invasive pulmonary biopsies enabling physicians to navigate deep into the peripheral airways to reach hard‐to‐access nodules for early detection of malignancy. Using a thin, steerable catheter guided by a thoracic surgeon/pulmonologist from a console, it offers greater precision and potentially fewer complications than traditional methods. It uses a patient's CT scan to plot a precise path and incorporates fiber optic shape‐sensing technology to navigate, enabling a new, improved method for diagnosing lung disease [1].

A thoracic surgeon/pulmonologist utilizes the instrument's software to create a 3D map of the patient's airways from a CT scan to identify the target lesion. A flexible robotic catheter is guided through the lung's airways, guided by real‐time imaging. Once the target lesion is reached, precise tissue sampling is obtained. Key benefits of this technology include. (1) A minimally invasive procedure that reaches peripheral lesions, previously inaccessible, reducing risk. (2) Enhanced precision as fiber optic shape‐sensing provides an accurate location and shape information of the target lesion. (3) Early detection and sampling of malignant nodules with improvement in treatment options. (4) Fewer potential complications including a pneumothorax leading to improved outcomes.

At our institution, the Ion robot is used routinely in combination with rapid on‐site evaluation (ROSE) in the evaluation of pulmonary nodules. Both FNAs and cryobiopsies are performed in the same setting, allowing for direct comparison of the two sampling methods. This study compares the diagnostic accuracy of the techniques.

## 2 Materials and Methods

### 2.1 Patients

From January 1, 2025 through June 30, 2025 the Cytopathology Laboratory of the University of Wisconsin Hospital and Clinics processed 87 fine needle aspirates (FNAs) obtained by Ion robotic assistance from 39 men, ranging in age from 37 to 83 years (mean, 67 years, median 68 years) and 40 women, ranging in age from 26 to 84 years (mean 68 years, median 69 years). Utilizing rapid on‐site evaluation (ROSE), aspirates were obtained from 22 right upper lobes, 7 right middle lobes, 17 right lower lobes, 24 left upper lobes and 14 left lower lobes. Cryobiopsies were performed on each nodule. The nodules ranged in size from 0.8 to 30 cm (mean 2.2 cm, median 1.9 cm) and greater than 50% were located peripherally.

### 2.2 Procedural Information

The pulmonary procedure was performed by four pulmonologists certified in the use of the Ion Endoluminal System (Intuitive Surgical, Sunnyvale, CA). A time‐out was performed with the patient prior to the start of the procedure. The patient's CT scan and 3‐D DICOM format were loaded onto the Ion computer. The target nodule was marked, segmented, and measured at 20 mm (mm). An airway plan was created using the automated software process. The information was stored in a jump drive and located on the Ion console in the operating suite.

The patient was placed in a supine position, anesthesia administered, and endobronchial intubation performed. A neuromuscular blockade was administered. The patient was intubated via the endotracheal tube (ETT) with the flexible video bronchoscope and the airways cleansed of mucus. The robot was locked to the swivel adapter on the ETT, the catheter was inserted into the ETT, and the accordion stabilizer was moved into position. The catheter was navigated to within 14 mm of the target nodule. Under fluoroscopy, the radial probe under endobronchial ultrasound was placed into the catheter, and the lesion was imaged showing that there was a concentric ultrasound image. Samples were then obtained for analysis. On average, 3 to 4 aspirates were obtained per nodule utilizing a 21‐gauge needle. From each pass, two slides were prepared. One slide was alcohol‐fixed for Papanicolaou staining, and the other slide was air‐dried and stained with Hema‐Diff stain for immediate adequacy assessment by a cytotechnologist. The cytotechnologist reviewed those cases that showed atypical cells to malignancy with the cytopathologist, and a preliminary diagnosis was provided. The needles were rinsed in Hank's balanced salt solution, and 1 to 3 additional passes were obtained for cell block preparation. On average, 2 to 4 transbronchial biopsies were obtained using a 1.1 mm cryoprobe with up to a 5 s freezing time. The sample pairs were taken concurrently with the FNA followed by the cryobiopsy in the same topographic area. The biopsies were fixed in 10% buffered formalin and sent to the histology laboratory in surgical pathology for routine processing and staining. Briefly, the biopsies were cut and stained with hematoxylin–eosin (H&E), as were the cell blocks.

The pulmonary aspirate and cell block slides were screened by five experienced cytotechnologists and signed out by five board certified cytopathologists. One subspecialty trained pulmonary pathologist and 2 experienced pathologists reviewed and signed out the pulmonary biopsies without knowledge of the cytology results. Cytohistologic correlation was conducted for this study. Discordant cases were reviewed by the author and pulmonary pathologist to assure diagnostic accuracy and reduce bias.

A comparable control cohort of 87 patients utilizing ROSE FNA and conventional transbronchial biopsy was compared to ROSE FNA and robotic‐assisted bronchoscopy data. The patient cohort (6‐month timeframe; January 1, 2023–June 30, 2023) was similar in age, pulmonary nodules aspirated, number of FNA passes, and biopsies performed. The study time period was chosen for analysis to ensure adequate usage and comfort of the pulmonologists with the new technology. The prior year, 2024, served as a transition year from use of the transbronchial techniques to the Ion robot.

### 2.3 Cytologic Reporting System

Cytologic diagnoses were classified as malignant, suspicious, neoplasm; other, negative, or nondiagnostic. Nondiagnostic samples were defined as those with no or scanty cellular material insufficient for analysis. A negative cytologic sample is synonymous with the absence of malignancy and cellular atypia and contained benign cellular material. An atypical cytologic sample contains cells with morphologic features beyond normal/reactive changes but is insufficient to classify them as suspicious/malignant. A suspicious cytologic sample contains cells with morphologic features that quantitatively and/or qualitatively fall short of a definitive diagnosis of malignancy. A malignant cytologic sample contains cells that show malignant cytologic features.

The results of the FNAs and cryobiopsies were confirmed by additional modalities including surgical procedures, radiologic studies, 6‐month clinical follow‐up and/or death from disease, which served as the gold standard for the final diagnosis. Sensitivity, specificity, and diagnostic yield (TP + TN/TP + TN + FN + ND) were calculated for FNAs and cryobiopsies.

For calculations, specimens diagnosed as malignant/suspicious and neoplastic were considered true positives if the final diagnosis was malignant and false positives if the final diagnosis was benign. Lesions diagnosed as atypical were considered true negatives if the final diagnosis was benign and false negatives if the final diagnosis was malignant. So too were the calculations for negative and nondiagnostic cases. For the purposes of this study, nondiagnostic cases were included in the calculations, as neoplastic cells were found on both FNAs and cryobiopsies. Surgical pathology reports were reviewed for all cryobiopsy specimens.

## 3 Results

**FIGURE 1:**
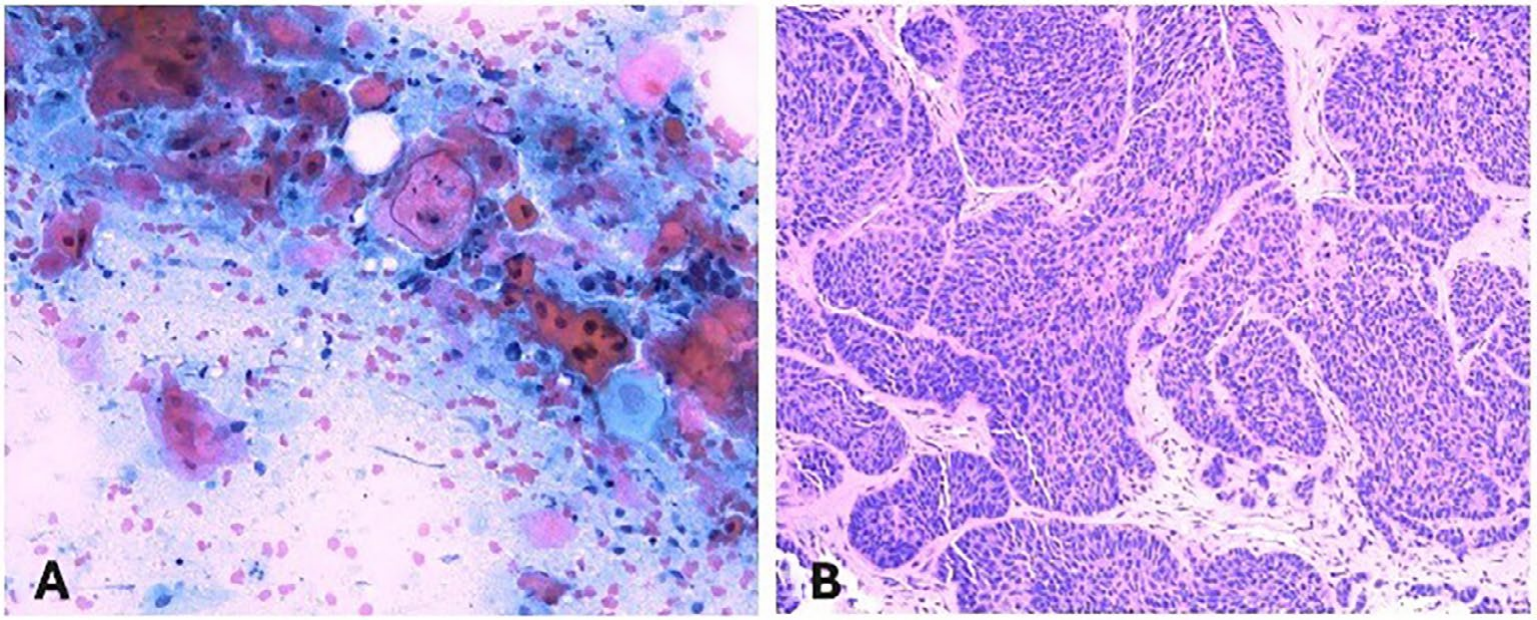
(A) Pulmonary FNA with clusters and single malignant squamous epithelial cells (Papanicolaou stain, 20×). (B) Concurrent concordant cryobiopsy with infiltrating squamous cell carcinoma (hematoxylin–eosin stain, 10×). [Color figure can be viewed at wileyonlinelibrary.com]

**FIGURE 2:**
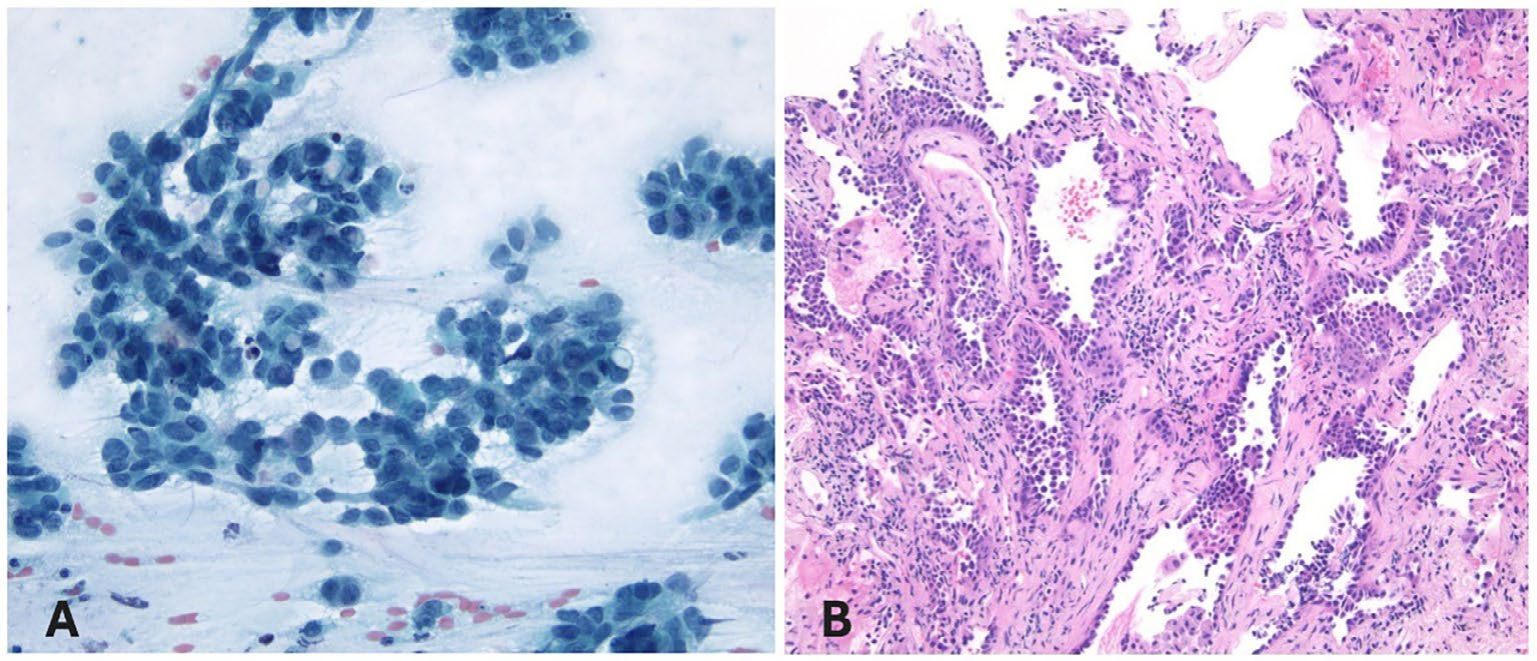
(A) Pulmonary FNA with clusters of adenocarcinoma cells (Papanicolaou stain, 20×). (B) Concurrent concordant cryobiopsy with infiltrating adenocarcinoma (hematoxylin–eosin stain, 10×). [Color figure can be viewed at wileyonlinelibrary.com]

In the current study (Table 1), 45 (52%)/87 FNAs were positive/suspicious for malignancy (13 nonsmall carcinomas, 9 adenocarcinomas, 9 squamous cell carcinomas, 2 poorly differentiated carcinomas; NOS, 1 small cell carcinoma, 2 metastatic melanomas, 2 metastatic prostate adenocarcinomas, 3 metastatic renal cell carcinomas). Further classification of the 13 nonsmall cell carcinomas showed 10 adenocarcinomas, 1 squamous cell carcinoma, 1 neuroendocrine carcinoma, and 1 atypical epithelial cell, insufficient for further evaluation on cryobiopsy. The 4 suspicious FNAs included 2 nonsmall carcinomas, 1 adenocarcinoma, and 1 suspicious for malignancy. Four (5%) FNAs were positive for a neuroendocrine (NET) tumor. Five (6%) were atypical, 13 (15%) were negative, and 20 (23%) were nondiagnostic. Cryobiopsies were concordant in 59 (68%) cases (Figures 1 and 2). Of the 59 concordant diagnoses, 46 (78%) were positive/suspicious for neoplastic cells; these included 43 (93%) primary and metastatic carcinomas, including 20 pulmonary adenocarcinomas, 12 squamous cell carcinomas, and 1 small cell carcinoma. Metastasis included 2 melanomas, 2 prostate adenocarcinomas, 3 renal cell carcinomas, 1 colon adenocarcinoma and I case of Hodgkin's disease. There were 3 (7%) NETs: 2 carcinoids and 1 neuroendocrine carcinoma. Thirteen (22%) of the 59 cases were negative and showed granulomas on both the FNAs and cryobiopsies.

**TABLE 1:** Cytohistologic correlation.

FNAs	Cryobiopsies						
	Nondiagnostic	Negative	Atypical	Neoplasm, other	Suspicious	Malignant	Total
Nondiagnostic	—	7	—	—	—	13	20
Negative	—	13	—	—	—	—	13
Atypical	—	—	—	—	—	5	5
Neoplasm, other	1	—	—	3	—	—	4
Suspicious	—	—	—	—	—	4	4
Malignant	1	—	1	—	—	39	41
Total	2	20	1	3	—	61	87

**FIGURE 3:**
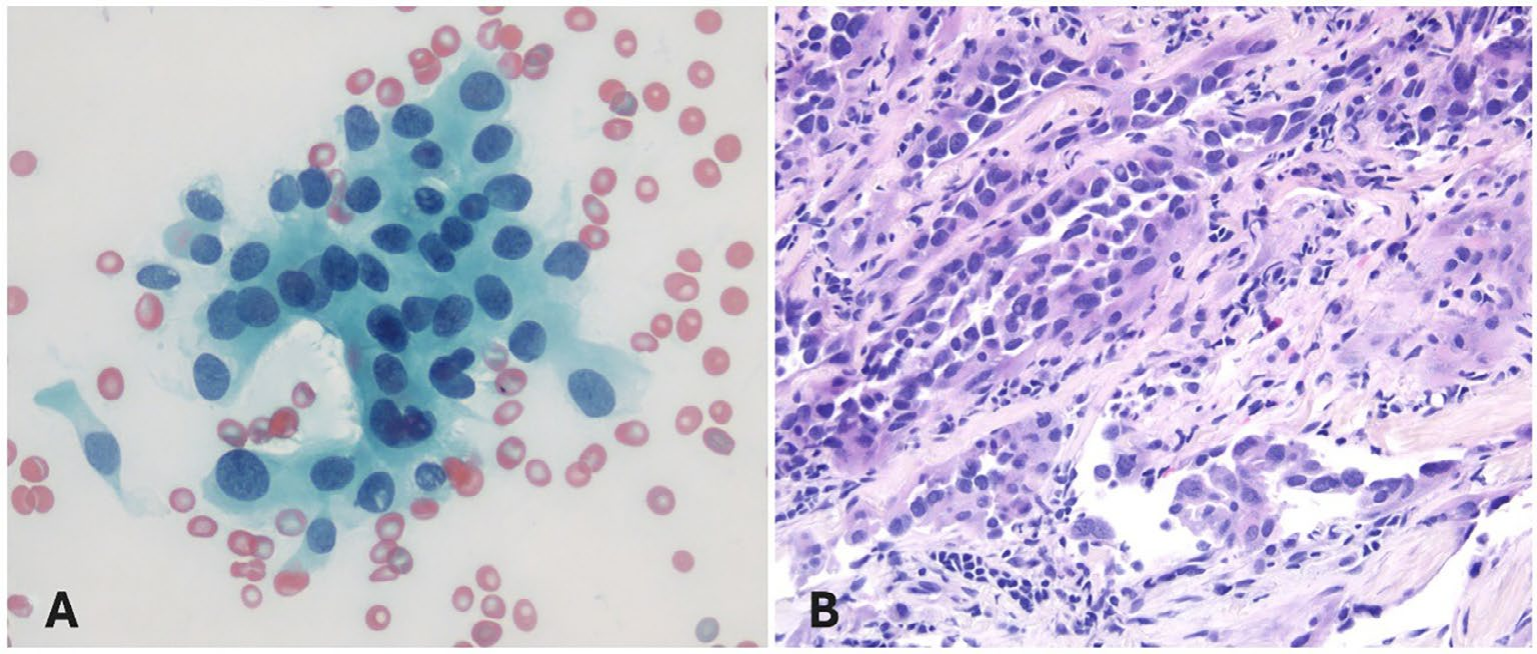
(A) Pulmonary FNA with a single cluster of atypical epithelial cells, NOS (Papanicolaou stain, 40×). (B) Concurrent discordant cryobiopsy with infiltrating adenocarcinoma (hematoxylin–eosin stain, 20×). [Color figure can be viewed at wileyonlinelibrary.com]

**FIGURE 4:**
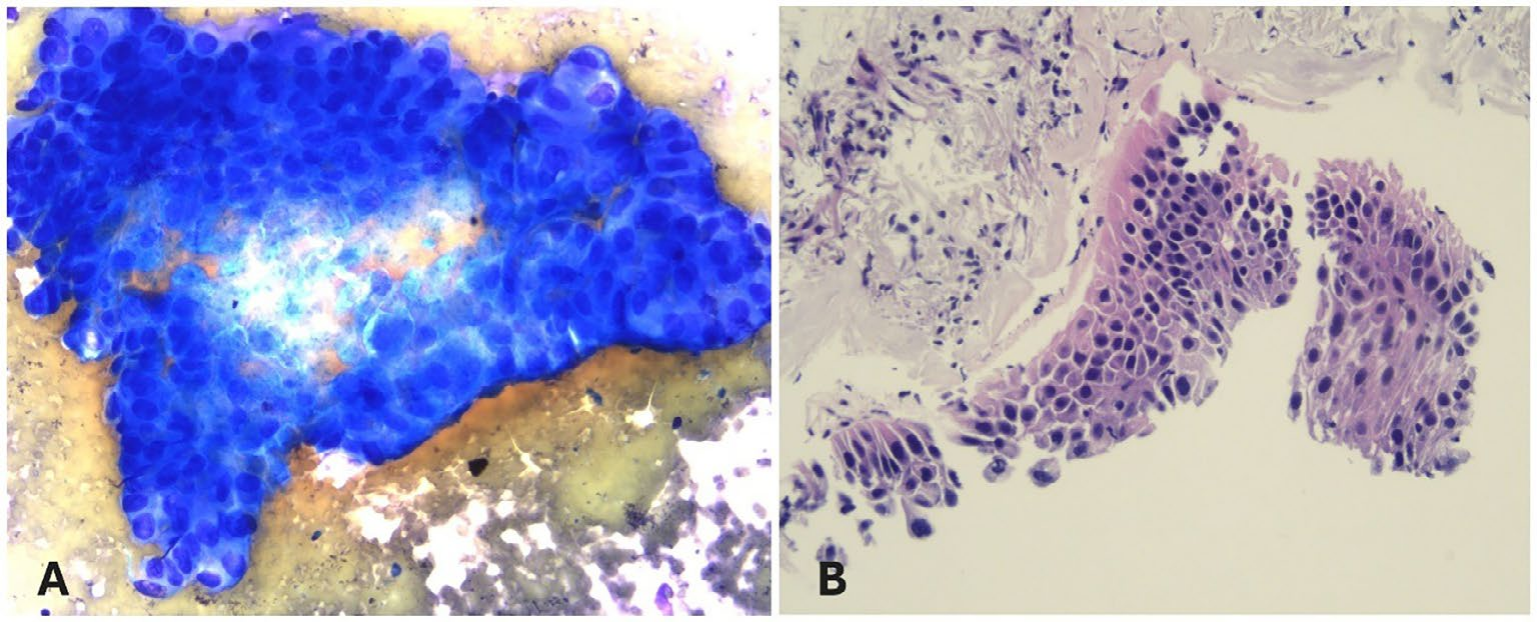
(A) Pulmonary FNA with a cluster of squamous carcinoma cells (Papanicolaou stain, 40×). (B) Concurrent discordant cryobiopsy with a minute fragment of detached atypical epithelial, NOS (hematoxylin–eosin stain, 20×). [Color figure can be viewed at wileyonlinelibrary.com]

Twenty‐eight (32%) cases were discordant (Figures 3 and 4). Of the 28 discordant cases, 3 (11%) had abnormal cells on FNA (1 neoplasm, other; 2 malignant), of which 2 were nondiagnostic and 1 showed atypical cells on cryobiopsy. Of the 5 (18%) with atypical cells, 3 showed adenocarcinoma on cryobiopsies, 1 showed squamous cell carcinoma, and 1 showed metastatic liposarcoma. Twenty (71%) nondiagnostic FNAs showed 4 adenocarcinomas, 4 squamous cell carcinomas, 1 small cell carcinoma, and 1 carcinoid tumor on cryobiopsies. Metastases included 1 renal cell carcinoma, 1 adenoid cystic carcinoma of the parotid gland, and 1 mucinous adenocarcinoma of the colon. Seven nondiagnostic FNAs showed granulomas, and 4 showed organizing pneumonia on cryobiopsies. Although cell blocks were made in each case, the 20 nondiagnostic FNAs had nondiagnostic cell blocks (23%).

The sensitivity for FNAs was 66% with a specificity of 100% and diagnostic yield of 71%. The sensitivity for the cryobiopsies was 94% with a specificity of 100% and a diagnostic yield of 96%. The overall difference between the two was statistically significant with a *p* < 0.001. Cryobiopsy demonstrated a statistically higher diagnostic yield and sensitivity for malignancy compared to FNA.

With regard to the control group, the sensitivity for FNAs was 75% with a specificity of 100% and diagnostic yield of 77%. The sensitivity of the transbronchial biopsies was 77% with a specificity of 100% and diagnostic yield of 78%. The overall differences between the two were not statistically different. However, higher diagnostic yields were obtained with cryoscopy usage as compared to transbronchial biopsies (96% vs. 78%, *p* < 0.001).

## 4 Discussion

Lung cancer is the leading cause of cancer death among men and women in the United States and the second most common malignancy reported as of 2024 [2]. Due to late‐stage diagnosis, the 5‐year survival rate is approximately 20% [3]. Early detection of pulmonary nodules has steadily increased as a result of increased screening programs and the greater use of computed tomography (CT) with an estimate of 1.6 million nodules detected annually [4].

Fine needle aspiration (FNA) is a safe, accurate, and minimally invasive procedure that has been utilized for decades in the work‐up and management of patients with pulmonary nodules. Advancements in ancillary testing, including immunohistochemistry and molecular analysis; however, require sufficient material that FNA alone may not always provide.

Flexible bronchoscopy has been instrumental in diagnosing pulmonary nodules but has been limited by the scope size, maneuverability, and the ability to easily pass tools through the bronchoscope [5]. In addition, diagnostic yields for sampling < 2 cm peripheral nodules is less than 50% [6]. Subsequently, transbronchial biopsy approaches have been utilized for sampling peripheral nodules to attain higher diagnostic yields [7] at the expense of a pneumothorax rate ranging between 18% and 25% [8]. Although electromagnetic bronchoscopy overcame the limitations of flexible bronchoscopy, the diagnostic yields remained lower than for transbronchial approaches [9]. Robotic‐assisted bronchoscopy platforms have recently gained wider acceptance for sampling peripheral pulmonary nodules. One of these platforms, the Ion Endoluminal System, received FDA approval in 2019 and is in use at our institution. The Ion robot, which uses shape‐sensing technology, allows for a more precise location of the nodule to be sampled and accurate sampling of the nodule over other technologies.

A comprehensive retrospective systemic review of the literature (2019–2024) reported on the performance of robotic‐assisted transbronchial biopsies [10]. Based on the study parameters which included robotic bronchoscopy, diagnostic yield, sensitivity and specificity, alone or in combination, 22 studies of which 17 utilized the Ion robot were reviewed. The number of transbronchial cryobiopsies ranged from 8 to 407 (mean; 117, median;110). The overall diagnostic yield was 87% with a sensitivity of 86% and a specificity of 99.7%. In a series of 324 nodules sampled by both FNA and cryobiopsy [11], FNA had a diagnostic yield of 70.4% and a sensitivity of 79.3%, as compared to cryobiopsy with a 92% and 90%, respectively (*p* < 0.001). Cryobiopsy demonstrated a statistically higher diagnostic yield and sensitivity for malignancy compared to FNA. The results of the current study support these findings.

Boac and colleagues [12] assessed the utilization of the Ion robot in the evaluation of 423 pulmonary nodules. The FNA diagnoses and the final diagnoses (a combination of clinical and cryobiopsy results) were concordant in 51% of the cases with a sensitivity of 66% and specificity of 100%. They concluded that the Ion robot was highly accurate but only moderately sensitive for an FNA diagnosis of malignancy. In addition, FNAs were not very accurate in the diagnosis of benign lesions. For example, 32 cases showed granulomas, of which only 4 (13%) were identified on FNA. In the current study, 7 nondiagnostic FNAs showed 3 granulomas and 4 showed organizing pneumonia on cryobiopsy.

At our institution ROSE is used routinely for the FNA assessment of pulmonary nodules. Cytotechnologists provide adequacy assessments prior to the performance of the cryobiopsy by the pulmonologist. Given the increased diagnostic yield of cryobiopsies, as shown in this study and others, is FNA necessary? The College of American Pathologists published guidelines on the collection and handling of thoracic small biopsy and cytology specimens for ancillary studies [13]. Based on the quality of the evidence in the literature, the strength of their recommendations ranged from low to strong. With regard to ROSE, the quality of the evidence was moderate. When posed the question “Should ROSE be used when performing endobronchial ultrasound‐guided transbronchial procedures (EBUS TBNA), if available”, the responses were mixed. Of the 238 responses, 75.2% agreed, but 13.45% disagreed. A number of comments raised the appropriate use of ROSE regarding its use at every procedure, as the needs vary depending on the clinical situation. Comments were made concerning cellular waste in slide preparation and the aspects of ROSE that can add as a barrier for its use. These include adequate personnel to cover ROSE, the time commitment for cytopathology personnel as well as pathologists with little reimbursement for this service. Although medical centers have more bandwidth to provide ROSE, many community hospitals may not.

The Ion robot has the flexibility to more precisely locate and sample peripheral nodules; in particular the upper lobes that are often difficult to access by conventional radiologic methods and less prone to the development of a pneumothorax. Cryobiopsies also acquire more diagnostic material than FNA for ancillary studies including immunohistochemistry, but more importantly material for molecular studies used in the management of patients.

In conclusion, robotic‐assisted bronchoscopy platforms provide an innovative approach to the sampling of pulmonary nodules. In the current study, the diagnostic yield of the Ion robot, 96%, surpassed that of FNA, 71%.

## Funding

The author has nothing to report.

## Ethics Statement

This study did not require ethical approval as it used anonymized data.

## Consent

The author has nothing to report.

## Conflicts of Interest

The author declares no conflicts of interest.

## Data Availability

Research data are not shared.
